# Ultra-Thin Highly Sensitive Electronic Skin for Temperature Monitoring

**DOI:** 10.3390/polym16212987

**Published:** 2024-10-24

**Authors:** Yuxin Wang, Yuan Meng, Jin Ning, Peike Wang, Yang Ye, Jingjing Luo, Ao Yin, Zhongqi Ren, Haipeng Liu, Xue Qi, Sisi He, Suzhu Yu, Jun Wei

**Affiliations:** 1 School of Materials Science and Engineering, Harbin Institute of Technology (Shenzhen), Shenzhen 518055, China; 21s055036@stu.hit.edu.cn (Y.W.); 23s155096@stu.hit.edu.cn (Y.M.); 23s155078@stu.hit.edu.cn (J.N.); 22s155067@stu.hit.edu.cn (P.W.); 21s155114@stu.hit.edu.cn (Y.Y.); 21b355005@stu.hit.edu.cn (J.L.); yinao@stu.hit.edu.cn (A.Y.); 20b955028@stu.hit.edu.cn (Z.R.); liuhaipeng@hit.edu.cn (H.L.); qixue@hit.edu.cn (X.Q.); 2 Shenzhen Key Laboratory of Flexible Printed Electronics Technology, Harbin Institute of Technology (Shenzhen), Shenzhen 518055, China; hesisi@hit.edu.cn; 3 School of Science, Harbin Institute of Technology (Shenzhen), Shenzhen 518055, China; 4 State Key Laboratory of Advanced Welding and Joining, Harbin Institute of Technology (Shenzhen), Shenzhen 518055, China

**Keywords:** temperature sensor, electronic skin, ultra-thin sensor, vanadium dioxide, PEDOT:PSS

## Abstract

Electronic skin capable of reliable monitoring of human skin temperature is crucial for the advancement of non-invasive clinical biomonitoring, disease diagnosis, and health surveillance. Ultra-thin temperature sensors, with excellent mechanical flexibility and robustness, can conformably adhere to uneven skin surfaces, making them ideal candidates. However, achieving high sensitivity often demands sacrificing flexibility, rendering the development of temperature sensors combining both qualities a challenging task. In this study, we utilized a low-cost drop-casting technique to print ultra-thin and lightweight (thickness: approximately 3 µm, weight: 0.61 mg) temperature sensors based on a combination of vanadium dioxide and PEDOT:PSS at room temperature and atmospheric conditions. These sensors exhibit high sensitivity (temperature coefficient of resistance: −5.11%/°C), rapid response and recovery times (0.36 s), and high-temperature accuracy (0.031 °C). Furthermore, they showcased remarkable durability in extreme bending conditions (bending radius = 400 µm), along with stable electrical performance over approximately 2400 bending cycles. This work offers a low-cost, simple, and scalable method for manufacturing ultra-thin and lightweight electronic skins for temperature monitoring, which seamlessly integrate exceptional temperature-measuring capabilities with optimal flexibility.

## 1. Introduction

Body temperature, a critical physiological signal indicating health status, can disrupt essential enzymes and cells when abnormal, potentially causing irreversible harm. Real-time and effective temperature monitoring thus plays a vital role in comprehending health conditions and detecting diseases early [1,2,3]. Conventional temperature monitoring methods, such as mercury and infrared thermometers, suffer from discontinuous measurement, cumbersome portability, and necessitate patients to remain still, thereby limiting flexibility. Electronic skins for temperature monitoring, capable of providing real-time, long-term, and accurate temperature monitoring with prompt data feedback, have garnered widespread research and attention [4,5,6].

Ultra-thin temperature sensors, owing to minimal thickness, facilitate rapid heat conduction, enabling faster temperature response and higher temperature accuracy [7,8]. This is vital for improving real-time monitoring efficiency, thus offering extensive application prospects in areas like wearable devices, medical instruments, and environmental monitoring [9]. Tröster et al. [10] encapsulated magnesium microstructures within a flexible polymer, fabricating biodegradable temperature sensors with a thickness of less than 56 µm. This device maintains stable electrical performance under a bending radius of 1.75 mm, with a temperature coefficient of 2.45 × 10^−3^ K^−1^. In order to reduce the sensor thickness to a few micrometers, Rogers’ group [11] utilized microlithographic techniques to fabricate serpentine-shaped temperature sensors on silicone elastic substrates, employing gold as the sensing material. However, this method involves intricate etching and transfer processes, making it unsuitable for large-scale industrial production. Furthermore, its sensitivity (0.103%) within the range of human skin temperature requires enhancement. Currently, there is limited literature on the fabrication of highly sensitive, micron-scale ultra-thin temperature sensors.

Transition metal oxides (such as vanadium dioxide, vanadium pentoxide, nickel oxide, and so forth) are commonly used thermistor materials, particularly exhibiting excellent temperature sensing capabilities in bulk or thin film forms [12,13,14]. However, they require high-temperature annealing for shaping or activation [15,16], limiting the choice of flexible substrates available. In our previous work [17], we employed a low-temperature spray-coating technique to print flexible temperature sensors based on a composite of vanadium dioxide (VO_2_) and poly(3,4-ethylenedioxythiophene) polystyrene sulfonate (PEDOT:PSS) on electrospinning substrates, achieving excellent thermal sensitivity (−2.712%/°C), high precision (85 mK), and ultra-fast response time (1 s) and recovery time (2.5 s). Nevertheless, the temperature sensor had a relatively large thickness (in the range of hundreds of micrometers). On the one hand, this thick sensor restricts reducing the bending radius to the micron level, thus limiting sensor application on the skin surface. On the other hand, the larger thickness implies longer heat transfer paths, resulting in greater temperature lag and diminished temperature accuracy. Therefore, to address these issues, we propose a strategy to print high-sensitivity, ultra-thin temperature sensors at room temperature on ultra-thin parylene substrates. In this work, to ensure high conductivity and enhance the bond between the thermistor material and the flexible substrate, we still utilized an ink composed of a combination of VO_2_ and PEDOT:PSS, rather than commonly used thermistor material like VO_2_ [18,19]. By drop casting ink onto a flexible parylene substrate deposited via vacuum vapor deposition, followed by parylene encapsulation, we created a sensor with a total thickness of just 3 µm and a weight of 0.61 mg. The ink drop-casting conditions for the thin substrates were precisely controlled. Compared to our previously published work, this sensor exhibits nearly double the thermal sensitivity (−5.11%/°C), over twice the precision (31 mK), and an order of magnitude improvement in both response time (0.36 s) and recovery time (0.36 s), enabling precise temperature monitoring. Furthermore, the sensor exhibits excellent mechanical flexibility, maintaining stable electrical performance through 2400 bending cycles (enhanced tenfold from our previous work), with a minimum bending radius of 400 μm (over 30 times superior to our previous work). Comfortably adhering to the surface of human skin, it clearly displays fingerprint information, showcasing its immense potential for real-time temperature monitoring as an ultra-thin electronic skin.

## 2. Experimental Section

### 2.1. Materials

PEDOT:PSS (1.5 wt.%, aqueous solution) and silver paster were purchased from Aladdin Reagent, Shanghai, China. VO_2_ nanoparticles (diameter: 100–200 nm) were purchased from Xi’an Qiyue Biotechnology Co., Ltd., Xi’an, China. Parylene was purchased from Beijing Sanji Reagent Technology Co., Ltd., Beijing, China.

### 2.2. Fabrication of Ink

VO_2_ powder was added to the mortar, and the pestle was rotated in a figure-eight pattern until the powder was uniformly ground. A certain amount of PEDOT:PSS water solution with a mass fraction of 1.5 wt.% was measured and diluted in 5 mL of deionized water. The VO_2_ powder was then mixed with the diluted PEDOT:PSS water solution and sonicated using an intelligent ultrasonic processor for 20 min to obtain a well-dispersed aqueous solution of mixed VO_2_ and PEDOT:PSS, forming the ink.

### 2.3. Fabrication of Temperature Sensor

Initially, the silicon wafer was subjected to alcohol ultrasonic treatment in an intelligent ultrasonic processor, followed by drying with a blow dryer. Subsequently, a dense and uniform parylene film was deposited onto the silicon wafer using vacuum deposition. Then, the flexible substrate underwent oxygen plasma treatment for 15 min to achieve a hydrophilic surface. Next, a uniform mixture of 3 µL, 6 µL, 9 µL, and 12 µL was dispensed onto the hydrophilic substrate surface using a pipette until complete ink evaporation. Afterward, a 200 nm thick silver electrode layer was deposited on the temperature sensing layer using an electron beam evaporation technique, with an electrode spacing of 200 µm. Copper wires were bonded to the silver electrodes using silver paste for subsequent sensor performance testing. Finally, another layer of uniform and dense parylene film was deposited onto the sensor using vacuum deposition to complete the sensor encapsulation. Notches were made along the edge of the silicon wafer using a blade, and the ultra-thin sensor was carefully peeled off the silicon wafer using tweezers to obtain the final ultra-thin temperature sensor.

### 2.4. Characterization

The morphology of VO_2_ nanoparticles was observed using a scanning electron microscope (SEM; Zeiss, Crossbeam 350 (Oberkochen, Germany)). The VO_2_ and PEDOT:PSS composite was characterized by X-ray diffraction spectroscopy using an X-ray diffractometer (XRD; D/MAX 2500 diffractometer (Rigaku, Japan)). The temperature of the sensor under heating and cooling cycles was measured using a homemade temperature control system. The electrical performance of the sensor was evaluated with an electrochemical workstation (CHI660e (Shanghai, China)). Mechanical bending loads were applied by a homemade bending device to characterize the electrical and mechanical properties of the sensors under bending conditions. Unless otherwise stated, all tests were carried out at room temperature of 25 °C and 65% relative humidity.

## 3. Results and Discussion

**Figure 1:**
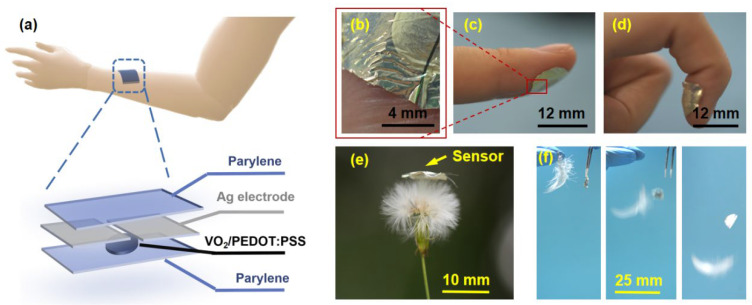
(**a**) Schematics of the structure of a high-sensitivity ultra-thin flexible temperature sensor. (**b**) Close-up diagram of the sensor on the index finger. (**c**,**d**) The sensor adheres well to the skin of the index finger when it is extended and bent. (**e**) The sensor is placed on a dandelion. (**f**) The sensor and the feather fall simultaneously under gravity.

Figure 1a illustrates the structure of the ultra-thin temperature sensor fabricated in this work. Initially, a flexible parylene substrate with a thickness of approximately 500 nm was prepared using the vacuum vapor deposition technique. The substrate underwent oxygen plasma treatment to enhance surface hydrophilicity [20], facilitating the adhesion of the parylene substrate to the thermistor material. Subsequently, VO_2_ nanoparticles and a solution of PEDOT:PSS were uniformly mixed in deionized water to create the ink. This ink was drop-cast onto the parylene substrate to form the thermistor material layer. Following this, silver electrodes with a spacing of 200 μm and a thickness of 200 nm were deposited via electron beam evaporation. Finally, a 500 nm thick parylene encapsulation layer was deposited using the vacuum vapor deposition technique to encapsulate the entire sensor. The thickness of the fabricated ultra-thin sensor is only around 3 μm.

VO_2_/PEDOT:PSS is the thermistor of the ultra-thin temperature sensor. VO_2_ is the main temperature-sensing material responding to temperature changes. PEDOT:PSS acts as a conductive agent and binder. The morphology and properties of the VO_2_/PEDOT:PSS composite were characterized by scanning electron microscopy (SEM) and X-ray diffraction (XRD) and reported in our previous work [17]. It was found that VO_2_ nanoparticles dispersed uniformly in the PEDOT:PSS and the peaks in the XRD pattern could be matched well to a standard PDF card (space group: P21/c, JCPDS No. 43-1051) of VO_2_ (M1). The VO_2_ and PEDOT:PSS in the composite will interact with each other [17], giving the thermistor a high-temperature coefficient of resistance.

The sensor’s minuscule thickness contributes to its outstanding flexibility and durability. These ultra-thin sensors can be conformally affixed to body parts necessitating temperature monitoring, such as the index finger. The sensor fits snugly against the skin, enabling clear visualization of fingerprint details without obstructing texture (Figure 1b). As depicted in Figure 1c,d, the sensor conforms to different states of the finger (straightening and bending) without any risk of breakage during movement. Moreover, the decreased sensor thickness translates to a lighter mass, weighing just 0.61 mg, which is virtually negligible. When placed on a dandelion, the sensor does not bend or distort the delicate dandelion fluff (Figure 1e). To illustrate its ultra-lightweight properties further, a comparison with a duck feather was conducted: both objects were simultaneously released from the same height, allowing gravity to take effect. Captured by a video camera, the sensor consistently falls below the duck feather, and over time, the gap between them widens (Figure 1f). Based on experimental findings, our developed ultra-thin temperature sensor proves to be lighter than a duck feather.

**Figure 2:**
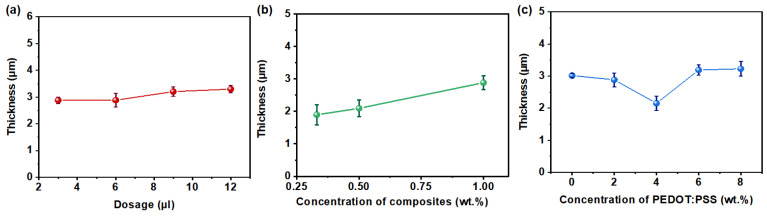
(**a**) Dependence of sensor thickness on ink drop coating dosage. (**b**) Dependence of sensor thickness on the concentration of VO_2_/PEDOT:PSS composite material in the ink. (**c**) Dependence of sensor thickness on the concentration of PEDOT:PSS in the VO_2_/PEDOT:PSS composite material.

To investigate the factors affecting sensor thickness for the preparation of ultra-thin sensors, we examined the influence of ink drop coating dosage, ink concentration, and ink composition on sensor thickness. The overall sensor thickness (approximately 3 µm) remains largely consistent regardless of the dosage of ink droplets dropped, as illustrated in Figure 2a. Additionally, Figure 2b illustrates the correlation between the concentration of VO_2_/PEDOT:PSS composite material in the ink and sensor thickness, showing an increase in thickness with higher concentrations of composite material in the ink. Further investigation into composite material composition revealed a trend where sensor thickness initially decreased and then increased with a rising concentration of PEDOT:PSS in the composite material, reaching its minimum thickness at a 4 wt.% PEDOT:PSS content (Figure 2c). In summary, optimal sensor thickness (about 3 µm) is attained with lower concentrations of composite material in the ink and an optimal PEDOT:PSS content in the composite material, while drop coating dosage has minimal impact on sensor thickness.

**Figure 3:**
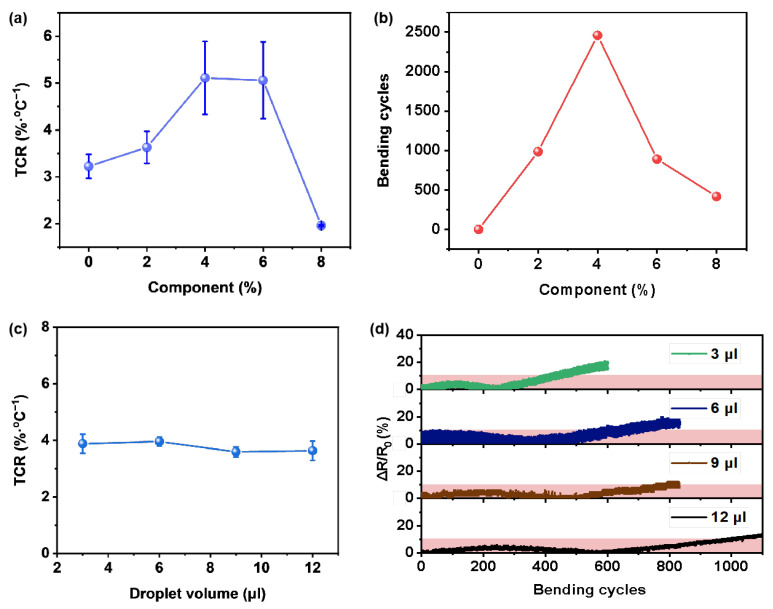
(**a**) Dependence of sensor temperature coefficient of resistance (TCR) on the concentration of PEDOT:PSS in the composite material. (**b**) Dependence of the maximum bending cycles (the relative resistance change is less than 10%.) of the sensor (bending radius = 17 mm) on the concentration of PEDOT:PSS in the composite material. (**c**) Dependence of the ink drop coating dosage on the sensitivity of the sensor. (**d**) Dependence of the sensor’s relative resistance change on the number of bending cycles (bending radius = 17 mm).

In pursuit of temperature sensors exhibiting exceptional temperature sensing capabilities, we first investigated the influence of PEDOT:PSS concentration in the composite material on the electrical and mechanical properties of the sensor. As illustrated in Figure 3a, by increasing the concentration of PEDOT:PSS in the composite material, the Temperature Coefficient of Resistance (TCR) of the sensor, typically reflecting temperature sensor sensitivity, initially increases before decreasing. It reaches peak values at PEDOT:PSS concentration of 4 wt.% and 6 wt.% (TCR > 5%/°C). Consequently, selecting inks with PEDOT:PSS concentrations of 4 wt.% and 6 wt.% will yield temperature sensors with superior electrical characteristics. To assess the durability of the temperature sensor under bending conditions, bending tests were conducted (Figure 3b). When the concentration of PEDOT:PSS is 0 wt.% (Figure S1), the sensor’s relative change in resistance exceeds 10% during the first bending cycle, reaching 20% by the sixth bending cycle, significantly surpassing the acceptable error range (where the relative resistance change is less than 10%). Consequently, without PEDOT:PSS added to the ink, the maximum number of stable bending cycles is zero, indicating poor electromechanical stability and rendering the sensor unsuitable for use under flexible bending conditions. Therefore, we increased the concentration of PEDOT:PSS in the composite material to bolster the sensor’s electromechanical stability during bending cycles. With a slight addition of PEDOT:PSS to the ink, the number of stable bending cycles increased rapidly. At a PEDOT:PSS concentration of 2 wt.%, the relative change in sensor resistance remained below 10% even after nearly 1000 bending cycles. Increasing the PEDOT:PSS concentration to 4 wt.% extended the number of bending cycles to almost 2500. These results indicate that incorporating a moderate amount of PEDOT:PSS into the ink enhances adhesion among discrete vanadium dioxide particles and between these particles and the flexible substrate, thereby improving the sensor’s electromechanical stability (Figure 3b). However, further increasing the concentration of PEDOT:PSS in the composite material results in a decrease in the maximum number of bending cycles for the sensor. After increasing the concentration of PEDOT:PSS to 6 wt.%, followed by a further rise to 8 wt.%, the number of stable bending cycles dropped below 1000 and subsequently fell below 500. Hence, considering the impact of the concentration of PEDOT:PSS on the mechanical and electrical properties of the sensor, we optimized it to 4 wt.%.

Following this, we conducted an investigation into the influence of varying ink drop coating dosages (3 µL, 6 µL, 9 µL, and 12 µL) on both the electrical and mechanical properties of the sensors. Remarkably, the temperature responses of the sensors exhibited similarity regardless of the ink drop coating dosages, as shown in Figure S2a–d. As temperature increased, sensor resistance decreased, and this trend reversed during cooling. Importantly, there was no noticeable temperature hysteresis in the resistance–temperature curves, which overlapped significantly during both warming and cooling. Moreover, the sensors demonstrated excellent sensitivity and linearity, with all four sensors achieving a calculated TCR surpassing 3.5%/°C and a linearity exceeding 0.97. Figure 3c presents the impact of ink drop coating dosage on the sensitivity of the sensor. Despite variations in ink drop coating dosage, the average TCR remained at 3.8%/℃ with a minimal deviation of only 0.16. This suggests that alterations in ink drop coating dosage have negligible effects on the sensitivity of the sensor. However, it was observed that varying ink drop coating dosages significantly influenced the electromechanical stability of the sensor, as illustrated in Figure 3d. Specifically, an increase in drop coating dosage correlated with a higher number of stabilized maximum bending cycles (bending radius = 17 mm). When the ink drop coating dosage increased to 12 µL, the maximum number of bending cycles for the sensor increased to 950. Consequently, for the production of flexible temperature sensors with superior resistance to mechanical bending, subsequent sensor preparation in this study utilized inks with a drop coating dosage of 12 µL and an optimized concentration of PEDOT:PSS of 4 wt.%.

**Figure 4:**
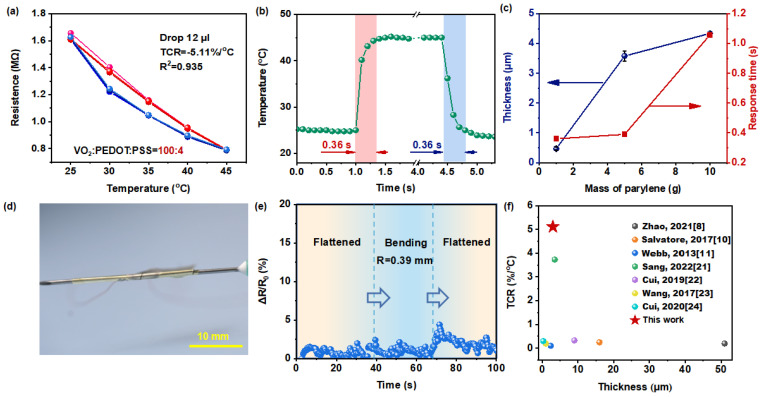
(**a**) Dependence of the resistance on temperature, with resistance measured every 5 °C step change in temperature and subjected to three consecutive heating-cooling cycles. (**b**) Response and recovery time from 25 °C to 45 °C. (**c**) Dependence of the response time and encapsulation layer thickness of the sensor on the quantity of parylene material used in vacuum vapor deposition. (**d**) A photograph of the sensor wound around a needle (bending radius = 400 µm). (**e**) The relative resistance change of the sensor in both flat and bent states. (**f**) Sensitivity and thickness comparison with state-of-the-art ultra-thin temperature sensors [8,10,11,21,22,23,24].

The performance of the optimized ultra-thin temperature sensor was evaluated. Initially, we assessed its sensitivity, which was found to be exceptionally high, with a TCR of −5.11%/^o^C, as shown in Figure 4a. Figure S3 illustrates the variation in sensor resistance during continuous heating to 45 °C followed by cooling to 25 °C (with temperature steps of 5 °C). The resistance exhibits a stepwise trend, decreasing with rising temperature and increasing during cooling. Across multiple heating and cooling cycles, the resistance pattern remains consistent with minimal differences, indicating the sensor’s stability in continuous temperature measurements and negligible temperature hysteresis effects. Figure 4b demonstrates that the sensor exhibits rapid response time (from 25 °C to 45 °C) and recovery time (from 45 °C to 25 °C), both measuring only 0.36 s, enabling timely temperature detection. Sequential testing examined the impact of varying encapsulation layer thicknesses on sensor response time (Figure 4c). As the quantity of parylene increased during vacuum deposition, the thickness of the encapsulation layer gradually increased from 0.5 μm to 4.3 μm, resulting in a corresponding increase in sensor response time. Thinner encapsulation layers offer shorter heat conduction paths, thus leading to shorter temperature response times. Therefore, we chose a thinner encapsulation layer (0.5 μm) to facilitate high-precision real-time tracking for body temperature monitoring. As a result, the sensor possesses outstanding temperature-sensing capabilities, allowing for precise and rapid temperature measurements. When the ultra-thin temperature sensor was wrapped around a syringe needle with a bending radius of 400 μm (Figure 4d), its resistance varied by less than 2.5%. Upon unwrapping from the needle, the resistance change was less than 5% (Figure 4e). In conclusion, the sensor demonstrates excellent mechanical flexibility, remaining stable and functional even under extreme bending conditions. Compared to a segment of published work (Figure 4f), our designed temperature sensor exhibits outstanding sensitivity and minimal thickness, making it more suitable for high-precision tracking in human skin temperature monitoring.

**Figure 5:**
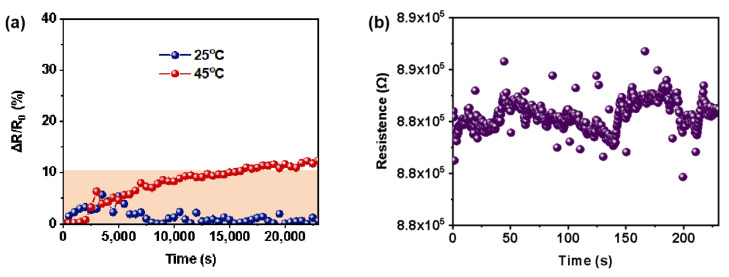
(**a**) Long-term stability to monitor resistance changes of the sensor at 25 °C and 45 °C. (**b**) Temperature measurement accuracy of the sensor in air (std. Dev = 0.031 °C).

Subsequently, a long-term stability test of the ultra-thin temperature sensor at low and high temperatures was carried out (Figure 5a). At room temperature (25 °C), the sensor displays remarkable stability, maintaining consistent resistance over a test duration exceeding 20,000 s. Under high-temperature condition (45 °C), stability is maintained for at least 15,000 s (the relative resistance change is less than 10%, within the permissible error range). Therefore, the ultra-thin temperature sensor could be reliably used in both low- and high-temperature environments over the long term.

Moreover, the long-term accuracy of the temperature measurement of the sensor was continuously monitored at 40 °C for 260 s (Figure 5b). It can be seen from the 600 data obtained that the resistance is consistent with little variations, resulting in a calculated temperature measurement accuracy of 0.031 °C.

**Figure 6:**
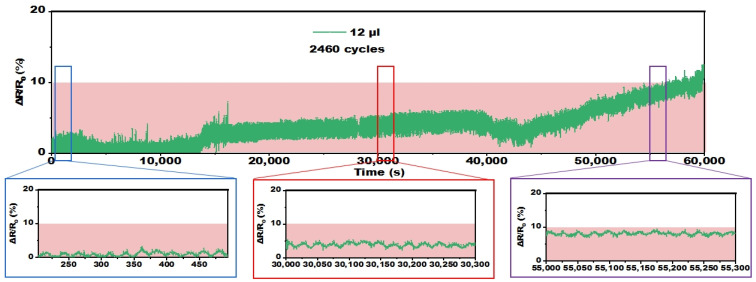
Dependence of resistance of the sensor on testing time during the cyclic bending test (bending radius = 17 mm).

Afterward, an analysis was conducted to assess the electromechanical durability of the optimized ultra-thin temperature sensor under bending condition (Figure 6). After 1200 and 2400 bending cycles, the resistance of the sensor increased by merely 5% and 10% respectively, remaining within the error tolerance. This indicates that the sensor maintains consistent electrical performance even after 2400 bending cycles, demonstrating outstanding mechanical durability.

## 4. Conclusions

In this study, we employed the drop-casting technique to fabricate an ultra-thin, highly sensitive vanadium dioxide-based electronic skin for temperature monitoring at room temperature. The sensor demonstrates high thermal sensitivity (−5.11%/°C), precision (31 mK), and ultra-fast response time (0.36 s) and recovery time (0.36 s), enabling precise temperature monitoring. Moreover, it possesses excellent mechanical flexibility, maintaining stable electrical performance within 2400 bending cycles, with a minimum bending radius of 400 μm. With a total thickness of only 3 µm and a weight of 0.61 mg, the sensor can comfortably and conformally adhere to the surface of human skin, displaying fingerprint information clearly. Our sensor opens up new opportunities and challenges for replacing traditional flexible sensor devices with ultra-thin electronic skins, thereby fostering further innovation in the Internet of Things.

## Data Availability

The original contributions presented in the study are included in the article, further inquiries can be directed to the corresponding authors.

## Supplementary Materials

The following supporting information can be downloaded at: https://www.mdpi.com/article/10.3390/polym16212987/s1, Figure S1. Dependence of the relative resistance change of the sensor on the testing time during the bending process (bending radius = 17 mm) when the relative content of PEDOT:PSS in the composite material is 0 wt.%. The right figure is a local enlargement diagram of the left one, Figure S2. Within the temperature range of 25 °C to 45 °C, the dependence of sensor resistance on temperature is investigated for (a) 3 µL drop coating, (b) 6 µL drop coating, (c) 9 µL drop coating, and (d) 12 µL drop coating, with resistance measurements taken every 5 °C. (The red curve indicates resistance variation during temperature increase, while the blue curve indicates resistance variation during temperature decrease), Figure S3. Dependence of the resistance of the sensor on time during continuous heating-cooling cycles within the temperature range of 25 °C to 45 °C.
