# Supplementary material for: Ultra-Thin Highly Sensitive Electronic Skin for Temperature Monitoring
Source: Polymers (Basel). 2024 Oct 24;16(21):2987. doi: 10.3390/polym16212987 (PMC11548264; doi:10.3390/polym16212987)
Supplement: Supplementary file 1 [file polymers-16-02987-s001.zip › polymers-3231299-supplementary.pdf]

Supplementary Material for:

Ultra-thin highly sensitive electronic skin for temperature monitoring

Yuxin Wang [1,2], Yuan Meng [1,2], Jin Ning [1,2], Peike Wang [1,2], Yang Ye [1,2], Jingjing Luo [1,2], Ao Yin [1,2], Zhongqi Ren [1,2], Haipeng Liu [1,2], Xue Qi [1,2], Sisi He [2,3], Suzhu Yu [1,2,*] and Jun Wei [1,2,4,*]

[1] School of Materials Science and Engineering, Harbin Institute of Technology (Shenzhen), Shenzhen 518055, China; 21s055036@stu.hit.edu.cn (Y.W.); 23s155096@stu.hit.edu.cn (Y.M.); 23s155078@stu.hit.edu.cn (J.N.); 22s155067@stu.hit.edu.cn (P.W.); 21s155114@stu.hit.edu.cn (Y.Y.); 21b355005@stu.hit.edu.cn (J.L.); yinao@stu.hit.edu.cn (A.Y.); 20b955028@stu.hit.edu.cn (Z.R.); liuhaipeng@hit.edu.cn (H.L.); qixue@hit.edu.cn (X.Q.)
[2] Shenzhen Key Laboratory of Flexible Printed Electronics Technology, Harbin Institute of Technology (Shenzhen), Shenzhen 518055, China; hesisi@hit.edu.cn
[3] School of Science, Harbin Institute of Technology (Shenzhen), Shenzhen 518055, China
[4] State Key Laboratory of Advanced Welding and Joining, Harbin Institute of Technology (Shenzhen), Shenzhen 518055, China
* Correspondence: szyu@hit.edu.cn (S.Y.); junwei@hit.edu.cn (J.W.)

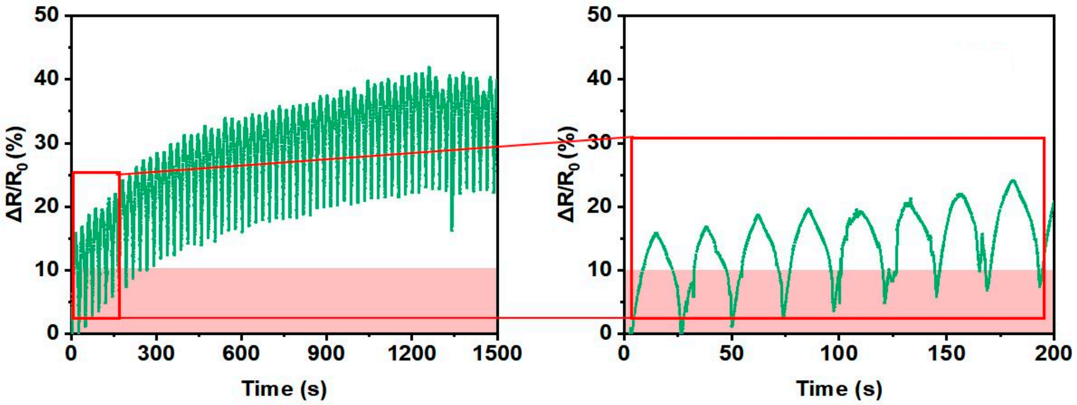

Figure S1. The dependence of the relative resistance change of the sensor on the testing time during the bending process (bending radius = 17 mm) when the relative content of PEDOT:PSS in the composite material is 0 wt.%. The right figure is a local enlargement diagram of the left one.

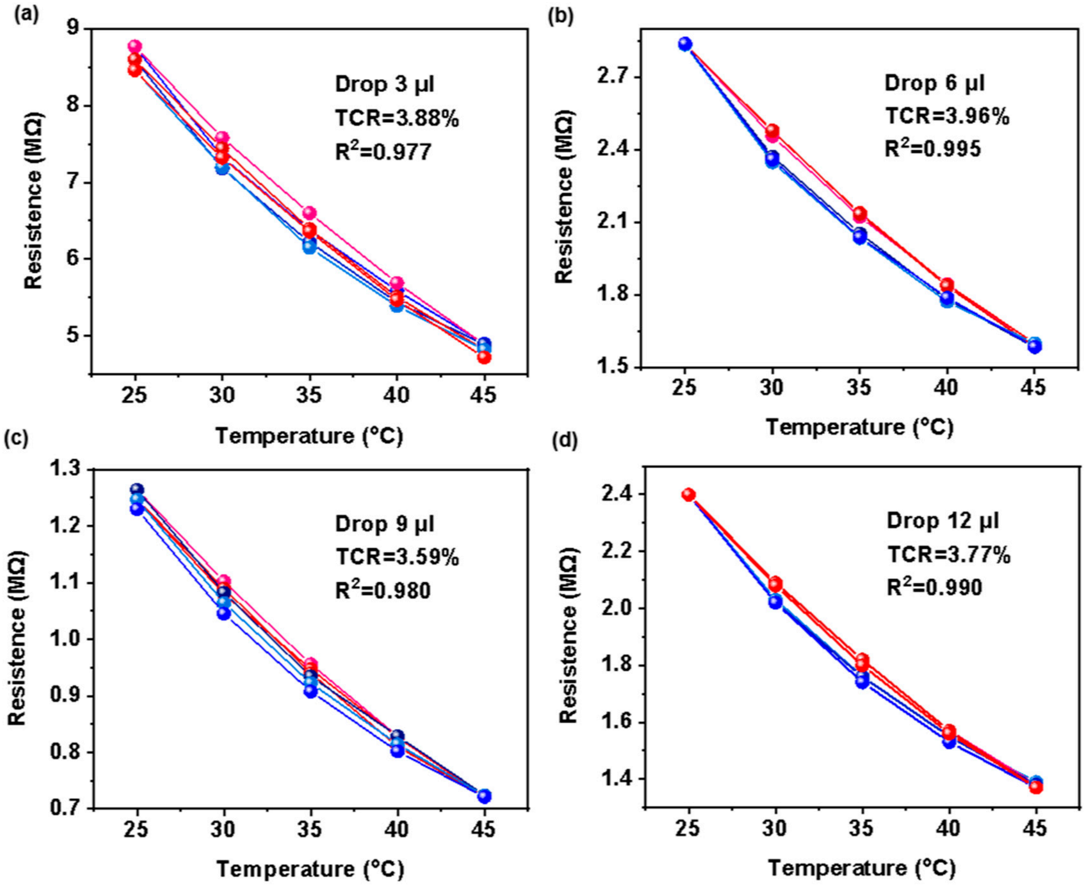

Figure S2. Within the temperature range of 25°C to 45°C, the dependence of sensor resistance on temperature is investigated for (a) 3 μl drop coating, (b) 6 μl drop coating, (c) 9 μl drop coating, and (d) 12 μl drop coating, with resistance measurements taken every 5°C. (The red curve indicates resistance variation during temperature increase, while the blue curve indicates resistance variation during temperature decrease.)

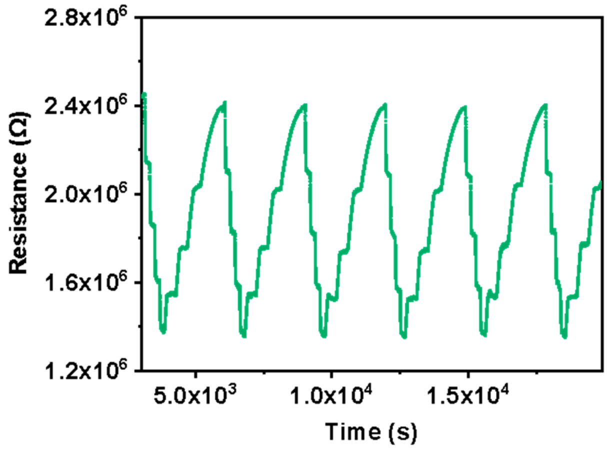

Figure S3. Dependence of the resistance on time during continuous heating-cooling cycles within the temperature range of 25ºC to 45ºC.
